# Inhibition of RANKL improves the skeletal phenotype of adenine-induced chronic kidney disease in mice

**DOI:** 10.1093/jbmrpl/ziae004

**Published:** 2024-01-14

**Authors:** Corinne E Metzger, Mizuho Kittaka, Alec N LaPlant, Yasuyoshi Ueki, Matthew R Allen

**Affiliations:** Departments of Anatomy, Cell Biology, and Physiology, Indiana University School of Medicine, Indianapolis, IN 46202, United States; Indiana Center for Musculoskeletal Health, Indiana University School of Medicine, Indianapolis, IN 46202, United States; Indiana Center for Musculoskeletal Health, Indiana University School of Medicine, Indianapolis, IN 46202, United States; Department of Biomedical Sciences and Comprehensive Care, Indiana University School of Dentistry, Indianapolis, IN 46202, United States; Departments of Anatomy, Cell Biology, and Physiology, Indiana University School of Medicine, Indianapolis, IN 46202, United States; Indiana Center for Musculoskeletal Health, Indiana University School of Medicine, Indianapolis, IN 46202, United States; Department of Biomedical Sciences and Comprehensive Care, Indiana University School of Dentistry, Indianapolis, IN 46202, United States; Departments of Anatomy, Cell Biology, and Physiology, Indiana University School of Medicine, Indianapolis, IN 46202, United States; Indiana Center for Musculoskeletal Health, Indiana University School of Medicine, Indianapolis, IN 46202, United States; Medicine/Division of Nephrology, Indiana University School of Medicine, Indianapolis, IN 46202, United States; Roudebush Veterans Administration Medical Center, Indianapolis, IN 46202, United States

**Keywords:** chronic kidney disease, cortical porosity, RANKL, PTH

## Abstract

Skeletal fragility and high fracture rates are common in CKD. A key component of bone loss in CKD with secondary hyperparathyroidism is high bone turnover and cortical bone deterioration through both cortical porosity and cortical thinning. We hypothesized that RANKL drives high bone resorption within cortical bone leading to the development of cortical porosity in CKD (study 1) and that systemic inhibition of RANKL would mitigate the skeletal phenotype of CKD (study 2). In study 1, we assessed the skeletal properties of male and female *Dmp1-cre RANKL^fl/fl^* (cKO) and control genotype (*Rankl^fl/fl^*; Con) mice after 10 wk of adenine-induced CKD (AD; 0.2% dietary adenine). All AD mice regardless of sex or genotype had elevated blood urea nitrogen and high PTH. Con AD mice in both sexes had cortical porosity and lower cortical thickness as well as high osteoclast-covered trabecular surfaces and higher bone formation rate. cKO mice had preserved cortical bone microarchitecture despite high circulating PTH as well as no CKD-induced increases in osteoclasts. In study 2, male mice with established AD CKD were either given a single injection of an anti-RANKL antibody (5 mg/kg) 8 wk post-induction of CKD or subjected to 3×/wk dosing with risedronate (1.2 μg/kg) for 4 wk. Anti-RANKL treatment significantly reduced bone formation rate as well as osteoclast surfaces at both trabecular and cortical pore surfaces; risedronate treatment had little effect on these bone parameters. In conclusion, these studies demonstrate that bone-specific RANKL is critical for the development of high bone formation/high osteoclasts and cortical bone loss in CKD with high PTH. Additionally, systemic anti-RANKL ligand therapy in established CKD may help prevent the propagation of cortical bone loss via suppression of bone turnover.

## Graphical Abstract

**Graphical Abstract:**
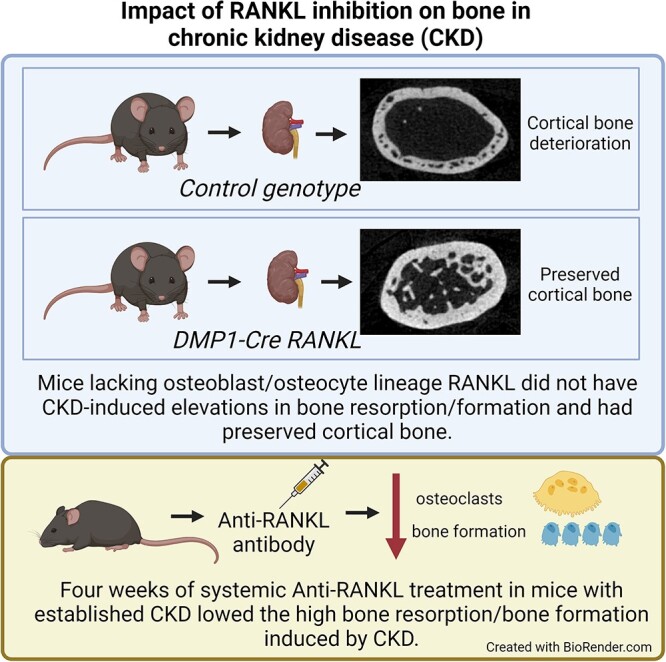

CKD is a progressive disease leading to a host of comorbidities including high rates of fracture in the clinical population.^1–4^ Cortical bone deterioration is a key characteristic of bone loss in CKD. Longitudinal assessment of CKD patients with high resolution computed tomography scans demonstrates rapid declines in cortical bone density, cortical area, and cortical thickness and increases in cortical porosity,^5^ all driven by high levels of bone resorption. In these patients, elevated PTH predicts changes in cortical bone although there is some heterogeneity across patients.^5^ Additionally, elevated PTH levels are also associated with increased risk of fracture in CKD patients.^6^ Although loss of cortical bone in CKD is linked to skeletal fragility, the mechanisms underlying cortical bone changes remain unclear.

Secondary hyperparathyroidism in CKD is associated with high bone turnover in clinical CKD patients^7^ and high formation/high resorption in multiple animal models of CKD.^8–11^ Along with high bone formation rate (BFR), osteoclastic bone resorption is high, likely contributing to the development of cortical porosity; however, what drives osteoclastic resorption within cortical bone to increase cortical porosity is not understood. Cortical porosity develops in both humans and in rodents with CKD,^5^^,^^7^^,^^8^^,^^12^^,^^13^ indicating the factors that drive porosity development may not be associated with traditional osteonal cortical bone structure/function since rodents do not normally have osteonal remodeling activity. Under normal physiological conditions, PTH acts directly on osteocytes, the most abundant bone cell that is embedded in bone, to increase RANKL which in turn increases osteoclastogenesis.^14–16^ We have previously shown that high PTH in adenine-induced CKD is associated with high osteocyte RANKL expression in cortical bone and cortical porosity,^9^^,^^12^ indicating osteocyte RANKL plays a role in the development of cortical porosity in CKD; however, the direct role of bone cell RANKL in CKD has not been assessed.

Treatment of CKD-induced bone loss is complex due to the multi-faceted manifestations of CKD, but targeting bone resorption with anti-resorptive therapy is logical due to the high osteoclastic stimulus in CKD. Systemic blockade of RANKL with denosumab is an approved antiresorptive therapy for osteoporosis and has been shown to rapidly decrease and sustain lower bone resorption markers while also decreasing bone formation markers to a lesser degree.^17^ In patients with mild and moderate CKD treated with denosumab, persistent gains in BMD and low incidence of fractures were noted with no differences in adverse effects across the stages of CKD assessed.^18^ Due to the high turnover in CKD, systemic blockade of RANKL is an attractive option to suppress bone turnover and reduce the high osteoclastic drive in CKD.

The goal of this current project was to assess the efficacy of targeting RANKL, both genetically and pharmacologically, on the skeletal manifestations of CKD. First, we utilized the DMP1-Cre model to primarily target osteocyte/osteoblast RANKL in mice with adenine-induced CKD to assess the development of high osteoclastic bone resorption and cortical porosity in the presence or absence of DMP-1-targeted RANKL (study 1). We hypothesized that a lack of bone-derived RANKL would decrease CKD-induced elevations in osteoclasts and prevent/reduce the development of cortical porosity. Second, we compared the impact of 2 different antiresorptive treatments, systemic blockade of RANKL and risedronate treatment, in mice with adenine-induced CKD on bone resorption/bone formation and cortical porosity after the development of porosity (study 2). We hypothesized that both pharmacological treatments would suppress CKD-induced high bone resorption and porosity.

## Methods

### Animals and timeline for study 1

*Rankl^fl/fl^* (018978) and *Dmp1-Cre* (023047) mice on the C57BL/6 background were obtained from the Jackson Laboratory) and were crossed to obtain *Dmp1-Cre Rankl^fl/fl^* mice (cKO) and littermate control mice (*Rankl^fl/fl^* mice; Con). Primers used for genotyping are Rankl_fl_F2: CTGGGAGCGCAGGTTAAATA^19^; 15 251 Rankl_fl_R1: AGCTTTTAGAATGCCAATAATTAAA (Jax); Rankl_fl_R2: GTCTAGGATAATGCACGGCACC.^20^ At 15 wk of age, half of the Con and cKO mice of each sex were assigned a control diet (CD) and were switched from the facility-provided grain-based diet to a purified casein-based diet with an adjusted calcium and phosphorus ratio with 0.9% phosphorous and 0.6% calcium (Teklad Diets [%TD.150303]; Inotiv). The remaining half of the Con and cKO mice were assigned adenine diet (AD) to induce CKD with the same casein-based diet with an addition of 0.2% adenine for 6 wk (Teklad Diets [TD.170948]; Inotiv). After 6 wk on the diet, AD mice were switched back to the control casein-based diet for the remaining 4 wk of the study. Previous work from our lab has demonstrated that a 6-wk induction period on a 0.2% AD sufficient to maintain reduced renal function and promote a CKD skeletal phenotype.^8^^,^^12^ Mice were monitored daily throughout the 10-wk protocol. All mice were injected with fluorochrome calcein labels 7 and 2 d prior to euthanasia. One female cKO AD mouse was found dead at 6 wk due to unknown causes (necropsy not performed), but all other mice made it to the study end point without complication. After 10 wk, animals were anesthetized via vaporized inhaled isoflurane and euthanized via exsanguination and thoracotomy. Right femurs were fixed in 10% neutral-buffered formalin for 48 h and then stored in 70% ethanol. All animal procedures were approved by the Indiana University School of Medicine Animal Use and Care Committee prior to the initiation of experimental protocols, and methods were carried out in accordance with relevant guidelines and regulations.

### Animals and timeline for study 2

Male C57Bl/6 J mice (JAX #000664; *n* = 48) were ordered from Jackson Laboratories at 15 wk of age and group housed 3–5 per cage at an institutionally approved animal facility with 12-h light/dark cycles. For this study, we utilized only males as we have previously shown similar skeletal phenotypes between male and female mice with adenine-induced CKD.^8^ At 16 wk of age, *n* = 12 mice were switched to the CD group for the remainder of the study as described above. The 3 adenine-induced CKD groups (AD; *n* = 12/group) were given the same diet with an addition of 0.2% adenine. After a 6-wk induction on the AD, all AD groups were switched back to the control casein-based diet for the remaining 6 wk of the study protocol. Beginning 8 wk following adenine induction, AD mice were divided into 3 treatment groups (*n* = 12/group)—untreated AD, AD+risedronate (AD+Ris), AD+anti-RANKL (AD+Anti-R). AD+Ris mice were given 1.2 μg/kg of risedronate (Sigma Aldrich) 3x/wk as a subcutaneous injection for 4 wk. This dose was selected as a lower, but more frequent dose than previously given^21^ to model a more frequent dosing scheme. AD+Anti-R mice were given a single dose (5 mg/kg) of an anti-RANKL monoclonal antibody (Oriental Yeast Company, Ltd, Bioindustry Division) as a subcutaneous injection. The 4-wk treatment time was chosen to match the timeframes of our previous work assessing treatments in established adenine-induced CKD.^21^^,^^22^ Mice were monitored daily throughout the 12-wk protocol (8 wk CKD induction +4 wk of treatment). All mice were injected with fluorochrome calcein labels 7 and 2 d prior to euthanasia. All mice made it to the study end point. After 12 wk, animals were anesthetized via vaporized inhaled isoflurane and euthanized via exsanguination and thoracotomy and tissues collected as described above. All animal procedures were approved by the Indiana University School of Medicine Animal Use and Care Committee prior to the initiation of experimental protocols, and methods were carried out in accordance with relevant guidelines and regulations.

### Serum biochemistries

Cardiac blood collected at time of euthanasia was used to measure serum blood urea nitrogen (BUN) via colorimetric assay as an assessment of presence/absence of kidney disease (BioAssay Systems). Serum 1–84 PTH was measured via ELISA (Immnotopics Quidel) with an intra-assay coefficient of variation of 2.4%–5.6% and an inter-assay coefficient of variation of 5.5%. Serum tartrate-resistant acid phosphatase 5b (TRAcP 5b) was measured as an assessment of systemic osteoclast numbers (Immunodiagnostic Systems). The intra-assay coefficient of variation for serum TRAcP 5b is 3.3%–6.9% and the inter-assay coefficient of variation is 5.2%–7.2%. All serum assays were run in duplicate with serum samples that had not been through any freeze/thaw cycles and followed all the procedures of the manufacturer.

### Micro-computed tomography

The right distal 1/3 femora were scanned 3 at a time as previously described^23^ on a SkyScan 1172 system (Bruker) with a 0.5 aluminum filter and an 8 μm voxel size. Trabecular bone was analyzed in a 1 mm region starting proximal to the growth plate in the distal femur. Cortical bone properties were analyzed on 5 contiguous slices located ~2.5 mm proximal to the growth plate in the distal femur. Cortical porosity was assessed from hand-drawn regions of interest on 5 contiguous slices, tracing the periosteal and endosteal surfaces. Bone volume was measured including the void spaces (pores) between the periosteal and endosteal surfaces. Cortical porosity was defined as the inverse of bone volume or the percent of void space within the cortical bone region (ie, 95% bone volume = 5% cortical porosity). A single average value from the 5 slices was obtained.

### Histomorphometry

Fixed undemineralized right distal femurs were subjected, after micro-CT scanning, to serial dehydration and embedded in methyl methacrylate (Sigma Aldrich). Serial frontal sections were cut 4 μm thick and left unstained for analysis of fluorochrome calcein labels. For trabecular measures, a standard region of interest of trabecular bone excluding primary spongiosa and endocortical surfaces was utilized. Total bone surface (BS), single-labeled surface (sLS), double-labeled surface (dLS), and interlabel distances were measured at 20× magnification. Mineralized surface to bone surface (MS/BS; [dLS+(sLS/2)]/BS × 100), mineral apposition rate (MAR; average interlabel distance/5 d), and BFR (BFR/BS; [MS/BS × MAR] × 3.65) were calculated. Intracortical analyses were completed on all adenine-induced CKD mice in study 2 (control animals and animal without cortical pores cannot be analyzed) within standardized regions that utilized both cortices (approximately 600 mm^2^ of tissue analyzed). The number of labeled pores, total labeled surface within pores, and interlabel distance was obtained. Calculations included pore number/bone area, MAR (interlabel distance/5) and BFR [100 × MAR × (label length/2)/bone area].

A second 4 μm-thick section was stained with tartrate-resistant alkaline phosphatase (TRAP) for the assessment of osteoclasts. For trabecular bone, sections were analyzed as osteoclast-covered trabecular surfaces normalized to total trabecular bone surface (Oc.S/BS, %) within the same region of interest as described above. For intracortical analyses within adenine mice in study 2, the osteoclast surface per bone surface within cortical pores (Oc.S/Pore Surface, %) was obtained.

To assess the impact of the treatments in study 2, a third 4 μm thick section was stained with Von Kossa/McNeal stain for the assessment of trabecular osteoid surfaces (OS/BS, %) and osteoid thickness (O.Th). These measurements were obtained in the same trabecular region of interest as described above. Adjusted apposition rate [Aj.AR; (MAR×(MS/OS)] and mineralization lag time [Mlt; (O.Th/Aj.AR)] were calculated. Histomorphometric analyses were performed using BIOQUANT (BIOQUANT Image Analysis). All nomenclature for histomorphometry follows the standard usage.^24^

### Statistical analyses

#### Study 1

All data were tested for normality with Levene’s test for equality of variances. If data met assumptions of normality, a 2×2 ANOVA (adenine-by-genotype) within sex was completed with main and interaction effects recorded. If the model ANOVA was statistically significant (*P* < .05), a Tukey post hoc was completed. If data did not meet normality, a Kruskal–Wallis test was completed. If the model *P*-value was statistically significant, pairwise comparisons were completed with Bonferroni correction for multiple tests. Main and interaction effects for normally distributed data or the *P*-value for the Kruskal–Wallis non-parametric test as well as post hoc analyses are reported on the graphs. All statistical analyses were completed with IBM SPSS Statistics 28 (IBM).

#### Study 2

All data were tested for normality with Levene’s test for equality of variances. If data met normality assumptions, a one-way ANOVA was completed with a Tukey post hoc if the model *P*-value was statistically significant. If the data did not meet normality assumptions, a Kruskal–Wallis test was completed with pairwise comparisons recorded. All statistical analyses were completed with IBM SPSS Statistics 28 (IBM).

## Results

### Study 1: Impact of DMP1-Cre RANKL deletion in adenine-induced CKD

#### Serum biochemistries

**Figure 1:**
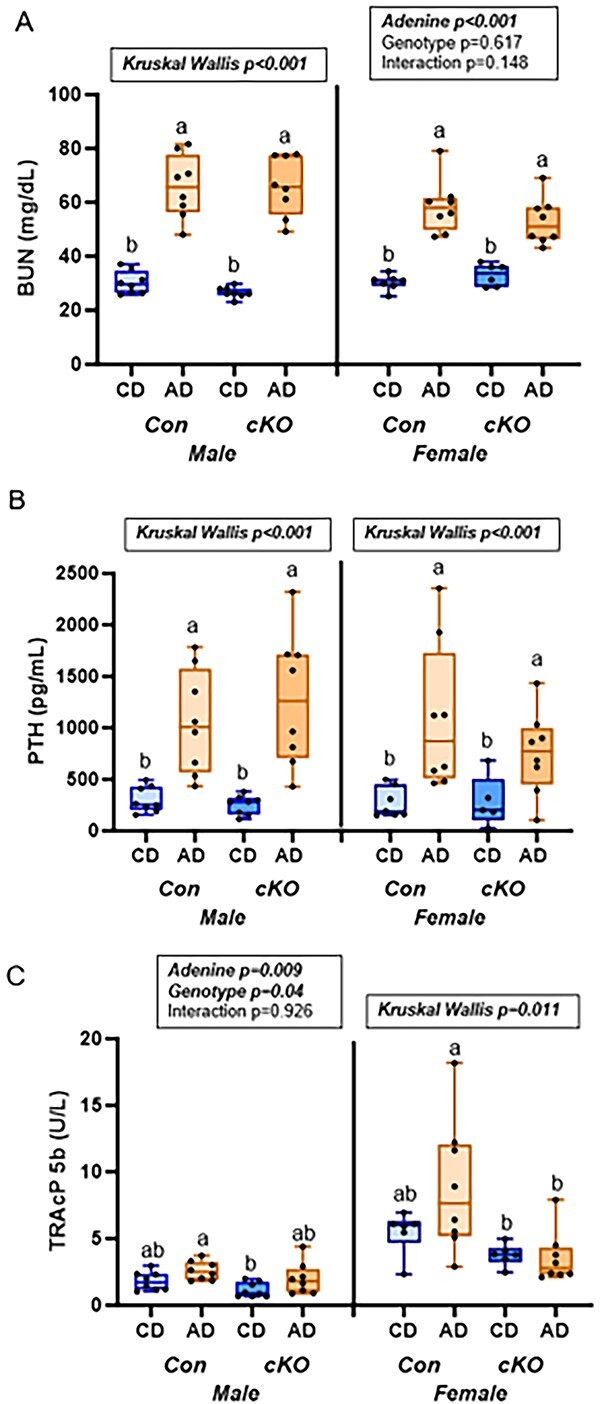
Serum markers from study 1—male and female mice, *Rankl^fl/fl^* (Con), and *Dmp1-Cre/Rankl^fl/fl^* (cKO) with dietary adenine-induced CKD (AD) and CD counterparts. (A) Serum BUN was higher in all AD mice. (B) Serum PTH was higher in all AD mice. (C) Serum TRAcP 5b trended higher in control genotype AD mice. Results of the 2×2 ANOVA or Kruskal–Wallis test are in the text box above the graph. Bars not sharing the same letters are statistically different (*P* < .05).

Both serum BUN and PTH in male mice were statistically different (*P* < .001 for both) with AD groups higher than CD groups regardless of genotype (Figure 1A). Similarly, in female mice, there was a main effect of adenine for serum BUN (*P* < .001) with AD mice having higher BUN than controls. Serum PTH in female mice was also higher in both AD groups compared to CD groups (Kruskal–Wallis *P* = .003). Overall, genotype did not lead to differing responses to adenine-induced CKD in BUN or PTH (Figure 1B). For serum TRAcP 5b in males, there was a main effect of genotype (*P* = .04) and a main effect of adenine (*P* < .001) with control genotype AD mice having the highest serum values of TRAcP 5b and cKO CD mice having the lowest group average. In females, serum TRAcP 5b was highest in the control genotype AD mice with both cKO having the lowest values (Kruskal–Wallis *P* = .011; Figure 1C).

#### Bone microarchitecture

**Figure 2:**
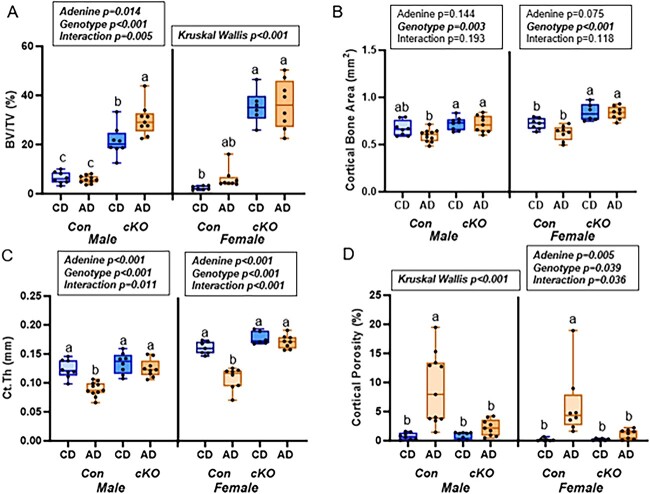
Distal femur bone microarchitecture from study 1—male and female mice, *Rankl^fl/fl^* (Con), and *Dmp1-Cre/Rankl^fl/fl^* (cKO) with dietary adenine-induced CKD (AD) and CD counterparts. (A) Both male and female cKO mice had higher trabecular bone volume than did Con mice. (B) Cortical bone area was lower due to AD in Con mice, but not different from CD in cKO mice. (C) Cortical thickness was lower in Con ADmice of both sexes, but cKO AD mice had preserved cortical thickness. (D) Cortical porosity was elevated in both male and female Con AD mice, but cKO AD mice had values not different from matched controls. Results of the 2×2 ANOVA or Kruskal–Wallis test are in the text box above the graph. Bars not sharing the same letters are statistically different (*P* < .05).

For trabecular bone volume at the distal femur in male mice, there was a main effect of both adenine (*P* = .014) and genotype (*P* < .001) with higher trabecular bone volume in cKO mice regardless of AD (Figure 2A). Additionally, cKO-AD mice had higher trabecular bone volume than Con-cKO control mice. Other trabecular bone variables are reported in Table 1. For cortical bone area, there was a main effect of genotype (*P* = .003) with both cKO groups higher than Con-AD (Figure 2B). Cortical thickness showed main effects of adenine (*P* < .001), genotype (*P* < .001), and an adenine-by-genotype interaction (*P* = .011) where cortical thickness was lower in the Con-AD group compared to all other groups (Figure 2C). Cortical porosity was not normally distributed (Kruskal–Wallis *P* < .001) with the Con-AD group having higher cortical porosity than all other groups (Figure 2D).

**Table 1:** Trabecular microarchitecture from the distal femur from study 1—male and female mice, *Rankl^fl/fl^* (Con), and *Dmp1-Cre/Rankl^fl/fl^* (cKO) with dietary adenine-induced CKD (AD) and control diet counterparts (CD). Groups not sharing the same letter are statistically different (*P* < .05).

**Sex**	**Genotype**	**Disease**	**Tb.Th (mm)**	**Tb.Sp (mm)**	**Tb.N (#/mm)**
Male	Con	CD	0.040 ± 0.004^b^	0.261 ± 0.030^a^	1.581 ± 0.458^b^
		AD	0.039 ± 0.003^b^	0.290 ± 0.021^a^	1.468 ± 0.319^b^
	cKO	CD	0.044 ± 0.004^b^	0.146 ± 0.031^b^	4.792 ± 1.197^a^
		AD	0.058 ± 0.007^a^	0.138 ± 0.017^b^	5.196 ± 0.807^a^
Female	Con	CD	0.037 ± 0.004^b^	0.324 ± 0.019^a^	0.630 ± 0.177^b^
		AD	0.042 ± 0.005^b^	0.301 ± 0.043^a^	1.440 ± 0.756^b^
	cKO	CD	0.080 ± 0.006^a^	0.178 ± 0.026^b^	4.410 ± 0.639^a^
		AD	0.082 ± 0.007^a^	0.184 ± 0.044^b^	4.389 ± 1.132^a^

**Figure 3:**
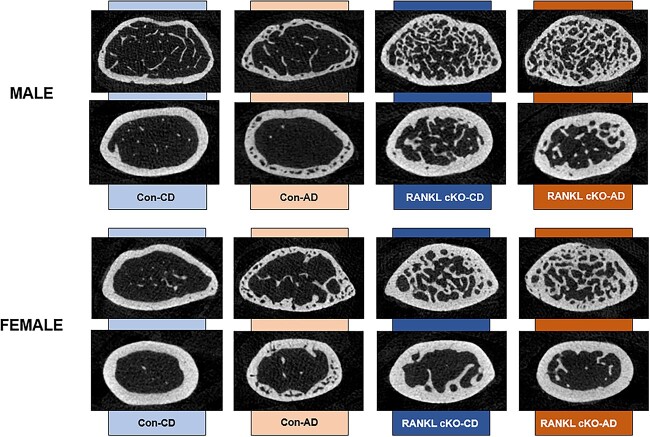
Representative images from distal femur micro-CT scans from male and female mice. The trabecular image (top panel within sex) is from the sample closest to the group mean for trabecular bone volume. The cortical image (bottom panel within sex) is the sample closest to the group mean for cortical porosity.

For female mice, trabecular bone volume was higher in cKO groups with Con-AD not different from any group (Kruskal–Wallis *P* < .001; Figure 2A). Other trabecular bone variables are reported in Table 1. For cortical bone area at the femur, there was only a main effect of genotype (*P* < .001) with both cKO mice having higher cortical bone area than both Con groups (Figure 2B). There were main effects of adenine (*P* < .001) and genotype (*P* < .001) and an adenine-by-genotype interaction (*P* < .001) in cortical thickness with Con-AD lower than all other groups (Figure 2C). Similar patterns were seen with cortical porosity with Con-AD higher than all other groups with main effects of adenine (*P* = .005) and genotype (*P* = .039) as well as an interaction effect (*P* = .036). (Figure 2D). Representative images of trabecular and cortical bone are presented in Figure 3.

#### Trabecular bone formation and osteoclast-covered surfaces

**Figure 4:**
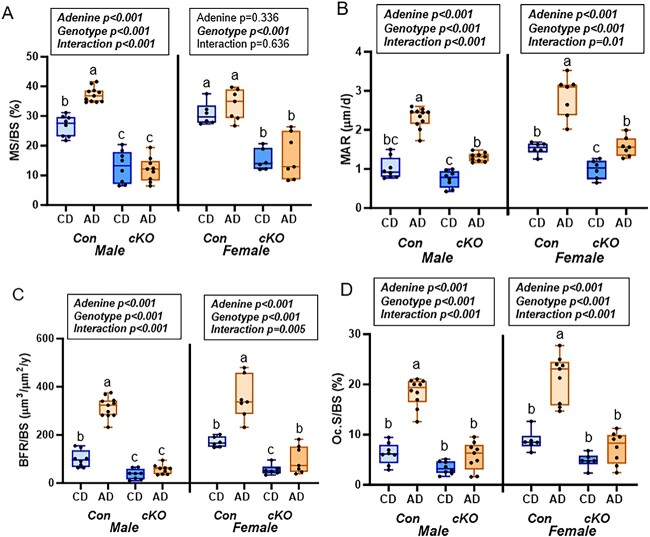
Histomorphometry of the distal femur trabecular bone in study 1—male and female mice, *Rankl^fl/fl^* (Con), and *Dmp1-Cre/Rankl^fl/fl^* (cKO) with dietary adenine-induced CKD (AD) and CD counterparts. (A) MS/BS higher in Con mice vs cKO mice. Male Con-AD mice had higher MS/BS than Con-CD. (B) MAR was elevated due to adenine in all groups of mice. (C) BFR was higher in Con-AD mice of both sexes. Female cKO-AD mice had higher BFR than matched cKO-CD mice.( D) Con AD mice of both sexes had elevated osteoclast-covered trabecular surfaces. cKO mice had no adenine effect on osteoclast surfaces. Results of the 2×2 ANOVA test are in the text box above the graph. Bars not sharing the same letters are statistically different (*P* < .05).

For male mice, there were main effects of adenine, genotype, and an adenine-by-genotype interaction for trabecular bone histological measures (*P* < .001 for all). For MSs, Con-AD had the highest group average followed by Con-CD with both cKO groups lower than all Con mice (Figure 4A). MAR was also highest in Con-AD followed by cKO-AD with Con-CD not different from either cKO group (Figure 4B). Con-AD mice had the highest BFR with both cKO mice having lower BFR than Con-CD (Figure 4C). Trabecular osteoclast-covered surfaces were higher in Con-AD mice compared to all other groups (Figure 4D). Con-AD mice had the highest number of osteoclasts compared to all other groups (Supplementary Figure S1). Supplementary Figure S1 shows the histological images of TRAP-stained trabecular bone surfaces.

In female mice, there were also main effects of adenine, genotype, and an adenine-by-genotype interaction for all trabecular bone histological measures except for mineralizing surfaces. For mineralizing surfaces, there was a main effect of genotype (*P* < .001), but no main effect of adenine (*P* = .336) nor an interaction effect (*P* = .636). Both Con groups had higher mineralizing surfaces than both cKO groups (Figure 4A). For MAR, Con-AD had the highest MAR with cKO-CD having the lowest MAR of all the groups (Figure 4B). Con-AD had the highest BFR followed by Con-CD and cKO-AD with cKO-CD having the lowest BFR (Figure 4C). The Con-AD group also had the highest osteoclast-covered surfaces of all groups (Figure 4D). For osteoclast numbers, there was a main effect of both genotype and adenine (*P* < .001 for both), but no interaction effect (*P* = .058). Con-AD mice had the highest number of osteoclasts followed by Con-CD mice and then both cKO groups (Supplementary Figure S1).

### Study 2: Comparison of antiresorptive treatments in adenine-induced CKD

#### Serum biochemistries

BUN was higher in all AD groups compared to the CD group (Kruskal–Wallis *P* < .001; Supplementary Figure S2). Serum PTH was also higher in all AD mice vs CD mice (*P* < .001; Supplementary Figure S2). Neither treatment, risedronate anti-RANKL, had an impact serum markers of kidney disease. For serum TRAcP 5b, the anti-RANKL-treated group had a higher group average than the other groups (*P* < .001; Supplementary Figure S2).

#### Bone microarchitecture

Trabecular bone volume was higher in CD mice compared to all AD mice (Kruskal–Wallis *P* < .001). Within treatment groups, AD+Anti-R was higher than AD, and the AD+Ris group was not different than either AD group (Table 2). Cortical bone area was different (*P* < .001) with CD higher than AD and AD+Anti-R and AD+Ris not different from any group (Table 2). Cortical thickness was also different (*P* < .001) with CD higher than all AD groups (Table 2). Similarly, cortical porosity was lower in CD mice compared to all AD groups (Kruskal–Wallis *P* < .001; Table 2).

**Table 2:** Trabecular and cortical microarchitecture from the distal femur from study 2—male adenine-induced CKD mice (AD) treated with risedronate (AD+Ris) or anti-RANKL (AD+Anti-R). Groups not sharing the same letter are statistically different (*P* < .05).

**Group**	**BV/TV (%)**	**Tb.Th (mm)**	**Tb.Sp (mm)**	**Tb.N (#/mm)**	**Ct.Ar (mm** ^**2**^ **)**	**Ct.Th (mm)**	**Ct.Po (%)**
***CD***	6.556 ± 1.472^a^	0.043 ± 0.005^a^	0.246 ± 0.019^c^	1.532 ± 0.351^a^	0.673 ± 0.067^a^	0.126 ± 0.011^a^	0.860 ± 0.466^b^
***AD***	3.626 ± 0.641^c^	0.035 ± 0.003^b^	0.316 ± 0.012^a^	1.029 ± 0.124^c^	0.543 ± 0.080^b^	0.093 ± 0.012^b^	4.593 ± 3.115^a^
***AD + Ris***	3.920 ± 0.558^bc^	0.034 ± 0.008^b^	0.308 ± 0.009^ab^	1.135 ± 0.111^bc^	0.604 ± 0.091^ab^	0.098 ± 0.010^b^	5.324 ± 1.980^a^
***AD + Anti-R***	4.753 ± 1.194^b^	0.038 ± 0.003^b^	0.297 ± 0.017^b^	1.239 ± 0.283^b^	0.552 ± 0.050^b^	0.091 ± 0.008^b^	3.810 ± 2.901^a^

#### Bone formation and osteoclast-covered trabecular surfaces

**Figure 5:**
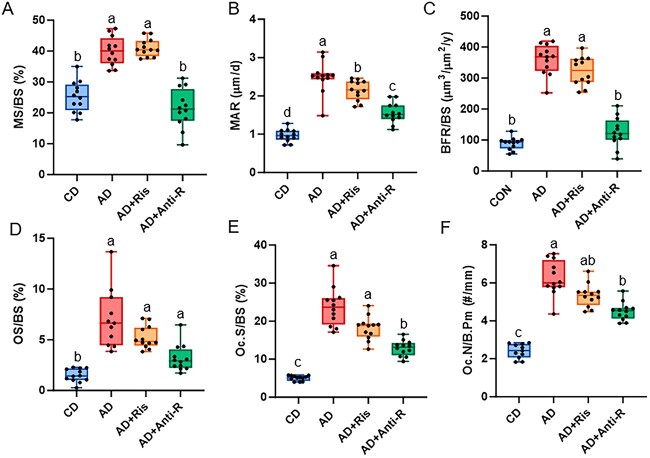
Histomorphometry of the distal femur in Study 2. (A) MS to BS was higher in AD and AD+Ris compared to CD and AD+Anti-R. (B) MAR was highest in untreated AD mice followed by AD+Ris then followed by AD+Anti-R with CD having the lowest MAR. (C) Untreated AD and AD+Ris had higher BFR than CD and AD+Anti-R. (D) Osteoid surface was higher in all AD groups compared to CD. (E) Osteoclast-covered trabecular surfaces were highest in AD and AD+Ris with AD+Anti-R statistically lower, but higher than CD. (F) Osteoclast numbers were highest in untreated AD with AD+Anti-R higher than CD, but lower than untreated AD. Bars not sharing the same letters are statistically different (*P* < .05).

Both mineralized surfaces (MS/BS) and MAR were statistically different (*P* < .001 for both) with AD and AD+Ris higher than CD and AD+Anti-R groups for MS/BS (Figure 5A). For MAR, the AD group had the highest MAR, followed by AD+Ris, followed by AD+Anti-R, and the control group had the lowest MAR (Figure 5B). Trabecular BFR was statistically different (*P* < .001) with AD and AD+Ris higher than the CD group and AD+Anti-R (Figure 5C). Importantly, the AD+Anti-R group was not different than the CD group. Overall, the anti-RANKL treatment mitigated adenine-induced increases in BFR through both a reduction in mineralizing surfaces and a decrease in MAR, while risedronate treatment only reduced MAR. Osteoid surfaces (OS/BS) showed that all AD groups had higher osteoid-covered surfaces than did control (Kruskal–Wallis *P* < .001; Figure 5D). A similar pattern was observed with osteoid thickness (*P* < .001 from a one-way ANOVA) with CD mice lower than all AD groups (Table 3). Both AJ.AR and mineralization lag time showed CD different from all AD groups (Kruskal–Wallis *P* < .001 for both; Table 3). Supplementary Figure S3 shows the histological images of osteoid. Overall, osteoid and mineralization calculations with osteoid and dynamic histomorphometry showed a clear effect of adenine, but no statistical differences with either treatment.

**Table 3:** Trabecular bone osteoid thickness, adjusted apposition rate, and mineralization lag time from study 2—male adenine-induced CKD mice (AD) treated with risedronate (AD+Ris) or anti-RANKL (AD+Anti-R). Groups not sharing the same letter are statistically different (*P* < .05).

**Group**	**Osteoid Thickness (μm)**	**Aj.AR (mcm/d)**	**Mlt (D)**
CD	1.380 ± 0.357^b^	24.783 ± 20.004^a^	0.087 ± 0.060^b^
AD	2.361 ± 0.645^a^	2.662 ± 0.546^b^	1.141 ± 0.546^a^
AD+Ris	2.538 ± 0.747^a^	3.843 ± 0.827^b^	0.714 ± 0.336^a^
AD+Anti-R	2.502 ± 0.586^a^	4.916 ± 2.591^b^	0.625 ± 0.329^a^

Trabecular osteoclast-covered surfaces were highest in AD and AD+Ris with the AD+Anti-R group lower than AD and AD+Ris but higher than CD (Kruskal–Wallis *P* < .001; Figure 5E). Osteoclast numbers were also different (*P* < .001) with untreated adenine having the highest value and anti-RANKL-treated mice statistically lower than AD, but higher than CD. The risedronate-treated group was not different from either AD group (Figure 5F).

**Figure 6:**
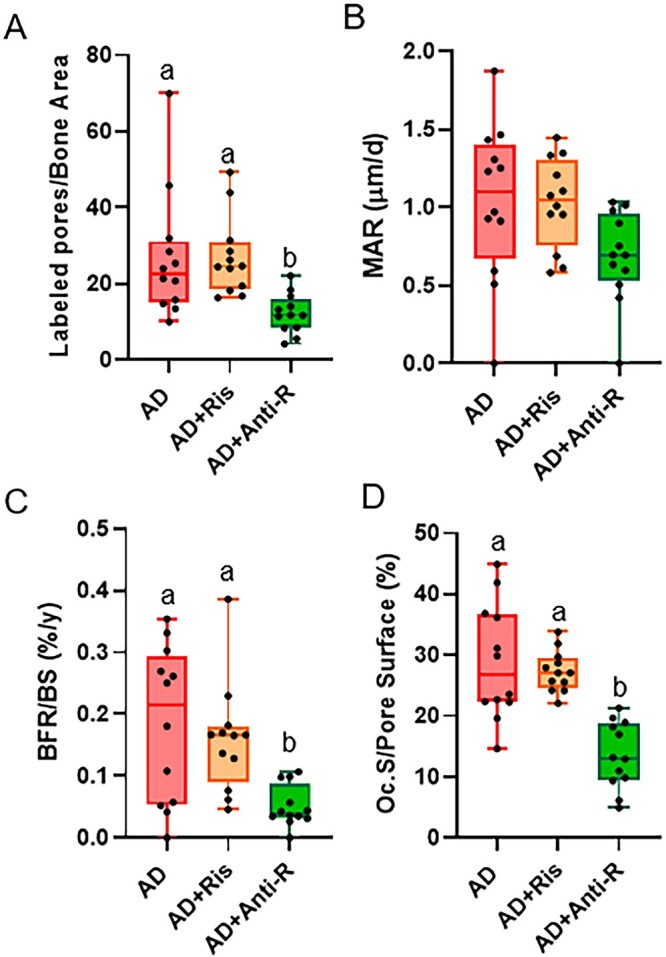
Intracortical remodeling in the distal femur cortical shaft in adenine mice from study 2: (A) anti-R-treated AD mice had lower labeled pores/bone area than other AD groups. (B) There were not statistical differences in MAR between adenine groups. (C) Anti-R-treated AD mice had lower intracortical BFR than other AD groups. (D) Osteoclast-covered pore surfaces were lower in anti-R-treated AD mice compared to other untreated and risedronate-treated AD mice. Bars not sharing the same letters are statistically different (*P* < .05).

For intracortical measures within the adenine groups, labeled pores/bone area was different between the 3 groups (*P* = .005) with the AD+Anti-R group lower than AD and AD+Ris (Figure 6A). MAR within cortical pores showed statistical difference (*P* = .044), but the Tukey post hoc did not show group differences (Figure 6B). Intracortical BFR within adenine groups demonstrated that AD+Anti-R was lower than the AD group and the AD+Ris group (Kruskal–Wallis *P* = .001; Figure 6C). Overall, the anti-RANKL treatment reduced intracortical BFR, while risedronate had no effect. Osteoclast-covered pore surfaces in the adenine groups also showed that AD+Anti-R group was lower than both other adenine groups (Kruskal–Wallis *P* < .001; Figure 6D).

## Discussion

These studies demonstrate that RANKL is an important driver of high bone turnover and cortical porosity in CKD with secondary hyperparathyroidism. Our first study demonstrated that *Dmp1-Cre* RANKL mice with adenine-induced CKD did not have a PTH-induced increase in bone resorption/bone formation and had preserved cortical bone despite no difference in serum PTH compared to control genotype adenine-induced CKD counterparts. This highlights the importance of bone-derived RANKL, primarily from the osteoblast/osteocyte lineage cells, in the development of high bone turnover and cortical porosity in CKD. Second, systemic inhibition of RANKL in mice with established CKD reduced bone formation and osteoclast numbers without impacting PTH. This was more effective than bisphosphonate (risedronate) therapy. Taken together, these studies indicate that methods to target RANKL should continue to be explored in scenarios where cortical porosity represents a clinical bone phenotype.

Cortical bone deterioration is a key component of bone loss in CKD. With longitudinal tracking via HR-pQCT scanning, average annual increases in cortical porosity changed most substantially compared to other trabecular and cortical properties measured at the distal radius and distal tibia.^5^ Animal models of CKD also show similar changes in cortical porosity and cortical thickness.^8^^,^^22^ In both cases, these skeletal changes are manifested through high rates of osteoclast bone resorption, either on the endocortical surface or intracortically. In this current work, control genotype adenine mice (*Rankl^fl/fl^*) had ~30% lower cortical thickness than non-adenine control genotype mice regardless of sex and both sexes had cortical porosity (average of ~10% porosity in males and ~6% porosity in females). Importantly, mice lacking osteocyte/osteoblast lineage RANKL (*Dmp1-Cre/RANKL^fl/fl^* mice) did not have adenine-induced CKD alterations to cortical bone; therefore, the lack of bone-derived RANKL via the *Dmp1-Cre* system prevented the declines in cortical thickness and progression of cortical porosity in CKD. All adenine mice of both genotypes had high BUN values, demonstrating the presence of CKD indicating that the differences in cortical bone are due to the genetic alterations in RANKL rather than alterations in renal function between genotypes.

Several other transgenic animal models have linked osteocyte lineage RANKL with cortical porosity. For example, the genetic suppression of apoptosis in the osteoblast/osteocyte lineage in aging mice had exaggerated cortical porosity and intracortical remodeling and elevated RANKL.^25^ In glucocorticoid-induced cortical bone loss in mice, osteocyte-specific RANKL deletion preserved cortical bone and prevented formation of porosity.^26^ These results are similar to what we found in our current study with CKD-induced cortical porosity development. Taken together, these studies allude to a central role of osteocyte/osteoblast produced RANKL in the initiation and development of cortical porosity loss across different conditions.

Secondary hyperparathyroidism in CKD patients is associated with cortical bone loss.^5^ In animal models, continuously high PTH in otherwise healthy animals is associated with cortical porosity development.^27^^,^^28^ In adenine-induced CKD in mice and in rats with progressive CKD due to polycystic kidney disease, high PTH is associated with cortical porosity development,^9^^,^^11^^,^^12^^,^^29^^,^^30^ Interestingly, the PTH response to CKD also differs due to the genetic strain of mice and these changes correlate with porosity development. For example, C3H/Hej mice develop adenine-induced kidney disease without elevated PTH and do not develop cortical porosity, while C57Bl/6 J mice, similar to this study, have high PTH and porosity development.^9^ Since PTH acts directly on bone cells to increase RANKL which stimulates osteoclastogenesis,^14–16^ RANKL is a logical link between high PTH and cortical porosity development. We and others have shown high RANKL in animal models of CKD.^9^^,^^12^^,^^31^ These current studies revealed that adenine mice, regardless of genotype, had 2-to-4-fold higher circulating PTH than sex-matched control groups; however, only control genotype adenine mice with intact RANKL signaling had cortical porosity development and loss of cortical bone. Therefore, our study demonstrates that RANKL, particularly from the osteocyte/osteoblast lineage cells, is essential for the cortical bone phenotype of high PTH-CKD.

Secondary hyperparathyroidism is also associated with high bone turnover, elevated bone resorption and bone formation, in both clinical patients with CKD^7^ and animal models of CKD.^8^^,^^9^^,^^11^^,^^32^ Suppression of PTH in animal models decreases both bone resorption and bone formation while also suppressing the formation of cortical pores.^10^^,^^11^^,^^22^ In our first study, control genotype adenine mice had 2.5–3-fold higher trabecular osteoclast surfaces than control genotype non-adenine mice regardless of sex, but *Dmp1-Cre* RANKL mice had no elevations in osteoclast surfaces due to adenine-induced CKD. For BFR, control genotype adenine-induced CKD mice of both sexes had 1–2-fold higher BFR than CD counterparts due to higher MS/BS and MAR in male mice and primarily due to higher MAR in female mice. In non-adenine *DMP1-Cre* RANKL mice, BFR was ~60%–70% lower than diet-matched control genotype mice showing a suppression of bone turnover due to genotype alone. In female mice, adenine-induced CKD led to ~80% higher BFR compared to the non-adenine counterpart, but *DMP1-Cre* RANKL male mice had no differences due to adenine within the genotype in BFR. Therefore, despite the high PTH, the *Dmp1-Cre/Rankl^fl/fl^* mice did not have a high bone formation/high osteoclast phenotype. Similarly, mice lacking osteocyte-derived RANKL given glucocorticoids did not have the typical increase in osteoclasts seen in wild-type mice and, as mentioned above, had preserved cortical bone.^26^ Therefore, these data continue to highlight bone-derived RANKL as a key link between continuously high PTH and the bone phenotype in CKD.

One limitation of this work is the growing understanding of the potential lack of specificity of the *Dmp1-Cre* approach to the osteoblast/osteocyte lineage and, therefore, possible non-bone effects of modulation of RANKL in our current model in other cells/tissues. For example, DMP-1 has been shown to be present in several extra-skeletal soft tissues in mice^33^ as well as other cells within bone, including bone lining cells.^34^ Additionally, *Dmp1-Cre* also marks skeletal muscle fibers, tissues within the brain, and gastrointestinal tissues.^35^ Therefore, we cannot conclude that only osteocyte lineage RANKL production was altered in the current study as other cells and potentially extra-skeletal tissues could also have been impacted. Recent work has shown that new technologies, like CRISPR interference, provide better cell-type specificity^36^ and could be valuable in future work to more fully isolate the effects of osteocyte RANKL within the context of CKD.

To bridge the translational gap of genetically modified animals to clinical therapy, we built upon the results from our first study by using anti-RANKL pharmacological therapy. We chose to compare anti-RANKL treatment compared to a more traditional anti-remodeling agent, risedronate, a member of the bisphosphonate class of therapeutics. In this study, our goal was not to prevent, but rather to mitigate or reverse the CKD bone phenotype. Previously, we found that a single dose of zoledronate worsened cortical porosity in animal models of CKD, while a weekly dose of risedronate had no effect on bone microarchitecture in adenine-induced CKD mice.^21^ Currently, we aimed to compare more frequent lower doses of risedronate (3x/wk) vs a single dose of an antibody against RANKL. We hypothesized that more frequent lower doses of risedronate compared to our previous study^21^ would be more effective at reducing osteoclastic drive into the cortical bone due to repetitive binding of the drug to the hydroxyapatite crystals on the bone surface and subsequent inactivation of osteoclasts. Like our previous study, we found no microarchitectural differences in bone due to risedronate treatment, but a 23% decrease in osteoclast-covered trabecular surfaces; however, it should be noted that osteoclasts in risedronate-treated bone may not all be active given the drugs’ mechanism of action. Anti-RANKL treatment, which acts by preventing osteoclastogenesis rather than inactivating osteoclasts at the bone surface, did result in greater trabecular bone volume than untreated adenine, but did not alter cortical bone parameters.

The lack of an effect of treatments on cortical bone structural parameters could be due to the length of the treatment. A longer treatment time potentially could result in a reversal of some of the CKD-induced bone structural changes, but it remains largely unclear how possible it is to reverse CKD-induced changes in bone. We have previously shown that the reversal of cortical pores is possible in animal models of CKD^22^ with similar treatment times to this study; however, it is important to note that we have only seen this with significant suppression of PTH beyond what is clinically achievable. Furthermore, utilizing a clinically relevant calcimimetic to suppress PTH in animal models did not result in such dramatic reversal of CKD-induced cortical bone changes.^37^ Therefore, we do not know if infilling of cortical pores/reversal of cortical thinning is possible with only the suppression of bone resorption, if longer treatment would be needed to see this effect, or if combination with PTH suppression is necessary for this to occur. Future studies will need to address these questions.

In our second study, importantly, we found that anti-RANKL treatment reduced BFR to values no different than the control group and reduced osteoclast-covered surfaces by 45% compared to untreated adenine. The elevated mineralization lag time seen in the adenine mice was not statistically different due to treatments, but the anti-RANKL-treated mice had ~45% lower mineralization lag time compared to untreated adenine, further indicating a reduction, though not complete normalization, of bone formation/mineralization. This suppression in the high bone formation/high osteoclast phenotype seen with non-specific anti-RANKL treatment was like the changes seen in the *Dmp1-Cre* RANKL mice. The mice in our second study had established CKD and, therefore, already had the presence of cortical pores, which is likely a more clinically relevant situation as CKD can progress silently for a considerable period of time. Intracortical analyses within adenine groups in our study demonstrated no effect of risedronate, but anti-RANKL treatment reduced both intrapore BFR and osteoclast-covered pore surfaces. Over time, we speculate that these changes could lead to both a reduction in the formation of new pores as well as a mitigation in existing pore development. Like our first study, systemic anti-RANKL treatment had no effect on circulating PTH levels which highlights that RANKL is a key link between high PTH and the bone phenotype in CKD. Additionally, our data allude to anti-RANKL treatment as a potentially more efficacious treatment for high bone turnover in CKD compared to bisphosphonate treatment.

Interestingly, we found a discrepancy between serum TRAcP 5b, a systemic marker that most clearly correlates with osteoclast numbers rather than resorption, and our histological assessment of osteoclast surfaces and numbers in the anti-RANKL-treated mice. We speculate that the tissue-specific measures showing suppression of osteoclast surfaces/numbers are more relevant in our model. Recent research has clearly shown that discontinuation of anti-RANKL treatment (denosumab) in human patients leads to increased bone resorption, bone loss, and increased fractures, particularly vertebral fractures.^38–41^ The timeline of this discontinuation/rebound effect in mouse models has not been clearly established, and it is unknown if this effect differs in a condition with high osteoclastic drive like CKD. Therefore, we do not know if the elevated TRAcP 5b in our anti-RANKL-treated adenine mice is in any way associated with a rebound effect. Regardless, it is clear within the distal femur trabecular and cortical bone that the dose and timing we utilized were efficacious in reducing osteoclast numbers. Future research would be important to address the potential timeline and rebound effect of cessation of anti-RANKL in CKD models.

In patients, a clinical goal has long been to lower elevated PTH levels; however, treatment with cinacalcet, a calcimimetic to lower PTH, did not significantly impact rates of clinical fracture.^42^ Both of our pre-clinical studies with adenine-induced CKD demonstrated positive changes in bone due to alterations in RANKL without changing PTH levels, indicating RANKL as an attractive potential therapy for mitigating bone changes in CKD. Small clinical trials utilizing denosumab, an antibody against RANKL, have shown reduced markers of bone turnover,^17^ reduction in fracture risk,^43^ and generally similar safety and efficacy in patients with mild and moderate CKD compared to those without CKD.^18^ In a small cohort of hemodialysis patients with severe secondary hyperparathyroidism, 6-months of denosumab treatment increased bone mineral density.^44^ In our mice that had established CKD, a single dose of anti-RANKL effectively mitigated high bone turnover and reduced the aberrant intracortical remodeling indicating the potential therapeutic benefits of systemic RANKL blockage even after the development of CKD-induced bone alterations. Our current study was not able to address whether anti-RANKL therapy alone could prevent or reverse cortical porosity that is already present at the start of treatment, but the reduced intracortical remodeling could mitigate the development of further porosity over time.

In conclusion, our studies identify the importance of RANKL as a driver of the CKD-induced bone phenotype. Bone-derived osteocyte/osteoblast lineage RANKL is associated with both the high bone formation/high osteoclasts observed in high PTH CKD as well as the deficits in cortical bone, both cortical thinning and formation of cortical porosity. Lack of bone-derived RANKL prevented CKD-induced bone microarchitectural and turnover alterations. Secondly, treatment with an anti-RANKL antibody after CKD was established, reduced both BFR and osteoclast surfaces on trabecular and pore surfaces without impacting PTH. Taken together these data demonstrate the importance of RANKL in the CKD bone phenotype and highlight its potential as a therapeutic target.

## Supplementary Material

Suppl_Fig_1_ziae004

Suppl_Fig_2_ziae004

Suppl_Fig_3_ziae004

## Data Availability

The data that support the findings of this study are available from the authors upon reasonable request.
