# Correction to: Inhibition of RANKL improves the skeletal phenotype of adenine-induced chronic kidney disease in mice

**DOI:** 10.1093/jbmrpl/ziae059

**Published:** 2024-05-08

This is a correction to: Corinne E Metzger, Mizuho Kittaka, Alec N LaPlant, Yasuyoshi Ueki, Matthew R Allen, Inhibition of RANKL improves the skeletal phenotype of adenine-induced chronic kidney disease in mice, *JBMR Plus*, Volume 8, Issue 2, February 2024, ziae004, https://doi.org/10.1093/jbmrpl/ziae004.

In the originally published version of this manuscript, there was an error in Figure 3, which showed a repetition of the male images in place of the female images. This error has been corrected.

The publisher apologises for the error.

