# Supplementary figures and images for: Inhibition of RANKL improves the skeletal phenotype of adenine-induced chronic kidney disease in mice
Source: JBMR Plus. 2024 Jan 14;8(2):ziae004. doi: 10.1093/jbmrpl/ziae004 (PMC10945718; doi:10.1093/jbmrpl/ziae004)

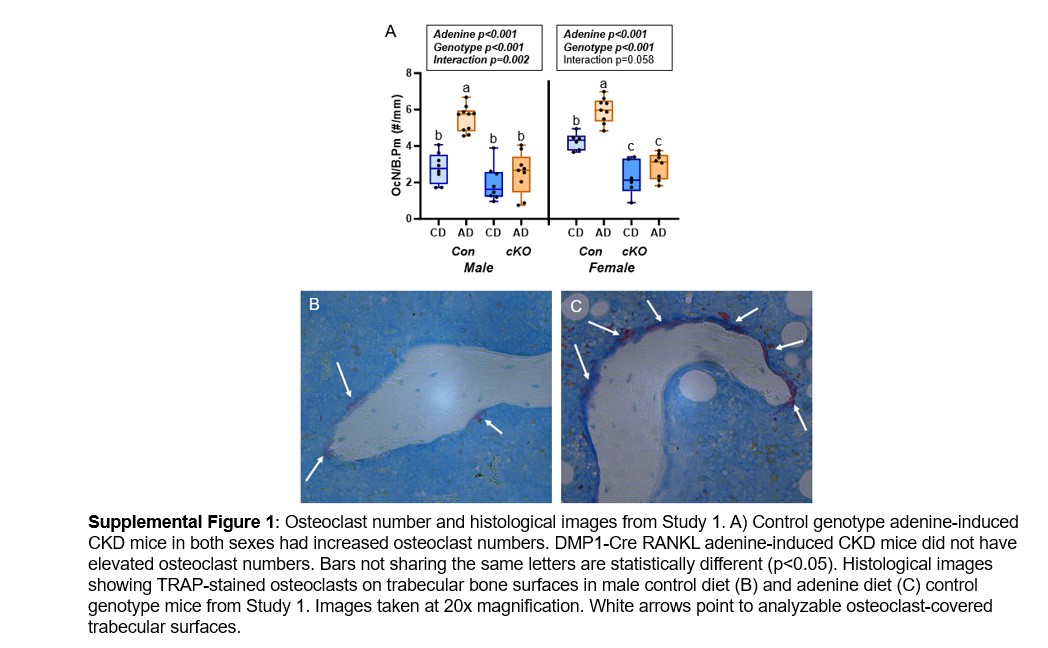

Supplement: Suppl_Fig_1_ziae004 [file suppl_fig_1_ziae004.jpeg]

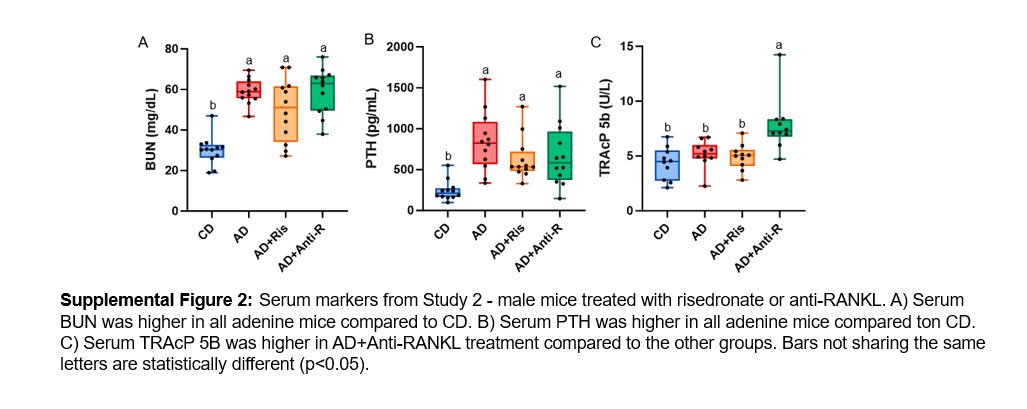

Supplement: Suppl_Fig_2_ziae004 [file suppl_fig_2_ziae004.jpeg]

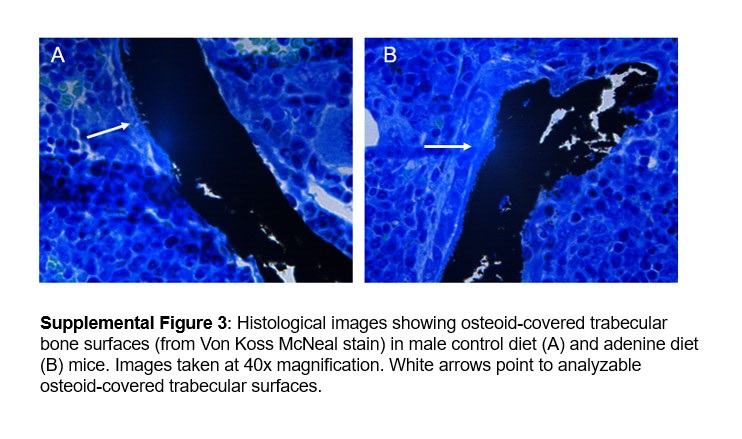

Supplement: Suppl_Fig_3_ziae004 [file suppl_fig_3_ziae004.jpeg]
